# Postoperative pain and surgical recovery: are we treating pain or building resilience?

**DOI:** 10.3389/fpain.2026.1835467

**Published:** 2026-06-03

**Authors:** Cecília Daniele de Azevedo Nobre, Bruno Augusto Parada, Bruno Vítor Martins Santiago, Hazem Adel Ashmawi

**Affiliations:** 1 Department of Anesthesiology, Rio de Janeiro State University, Rio de Janeiro, Brazil; 2 Department of Anesthesiology, Universidade Federal do Rio de Janeiro, Rio de Janeiro, Brazil; 3 Department of Anesthesiology, Universidade de São Paulo, São Paulo, Brazil

**Keywords:** pain modulation, patient-centered outcomes, perioperative care, postoperative recovery, prehabilitation, surgical resilience

## Abstract

Postoperative recovery remains highly variable despite advances in surgical techniques and perioperative care. Contemporary strategies, including Enhanced Recovery After Surgery (ERAS) protocols and multimodal analgesia, have improved outcomes but do not fully explain interindividual differences in recovery trajectories. Current perioperative models emphasize stress attenuation and symptom control but insufficiently address the mechanisms underlying the transition from acute postoperative pain to chronic pain and long-term disability. Surgical resilience is conceptualized as a dynamic, multidimensional adaptive process reflecting the patient's capacity to maintain or regain functional, psychological, and physiological equilibrium in response to surgical stress over time. Rather than a fixed trait, it emerges from the interaction between biological reserve, psychological adaptability, and behavioral engagement, modulated by contextual factors such as social support and access to care. Within this context, postoperative pain is not merely a symptom but a modifiable factor influencing recovery through physiological and behavioral mechanisms. In this perspective article, we emphasize the implementation of a multidimensional model integrating biological, psychological, behavioral, and clinical domains, in which pain management acts as a central modulator of recovery trajectories. This perspective highlights a critical gap in perioperative research and supports the transition toward personalized, resilience-oriented strategies.

## 1 Introduction

Despite remarkable advances in surgical techniques, anesthetic care, and perioperative monitoring, postoperative recovery remains highly variable among patients. Contemporary perioperative strategies have reduced surgical stress and improved outcomes; however, they do not fully address a central determinant of recovery—the patient's capacity to adapt to surgical insult. Perioperative care has evolved from a procedure-centered model to a broader, evidence-based approach incorporating multimodal analgesia, early mobilization, and structured recovery pathways (1, 2).

Programs such as Enhanced Recovery After Surgery (ERAS) have consistently reduced complications and hospital length of stay (3, 4). Nevertheless, patients undergoing similar procedures within optimized protocols often exhibit markedly different recovery trajectories, highlighting the limitations of models focused primarily on standardization rather than individual adaptive capacity (4).

Although concepts such as frailty, prehabilitation, and the interaction between pain, analgesia, and recovery have gained attention (5–7), their integration into a unified conceptual framework remains limited. In particular, surgical resilience has not yet been systematically incorporated into perioperative care models. At the same time, pain research has largely emphasized nociceptive mechanisms and pharmacological control, often overlooking psychological and behavioral determinants of recovery (8).

Emerging evidence suggests that psychological resilience is associated with improved postoperative outcomes, including reduced pain interference, faster functional recovery, and better quality of life (9, 10). However, conceptual heterogeneity and variability in measurement approaches limit its clinical applicability (10).

Importantly, resilience plays a critical role in modulating the transition from acute postoperative pain to chronic postsurgical pain. Emerging evidence demonstrates that patients with higher resilience exhibit reduced pain interference, lower analgesic requirements, and improved long-term functional outcomes. Conversely, low resilience is associated with maladaptive coping, increased pain catastrophizing, and higher risk of persistent pain and disability. Within this framework, resilience-oriented perioperative care represents a promising strategy not only to optimize short-term recovery but also to prevent chronic pain and long-term functional impairment (11).

This manuscript aims to discuss surgical resilience as a relevant construct in the perioperative context, exploring its conceptual foundations, the available tools for its measurement, and the possibilities for its operationalization in clinical practice. In this context, it seeks to reinforce the adoption of multidimensional models integrating biological, psychological, behavioral, and clinical domains as a means to better understand and improve postoperative recovery trajectories.

### 1.1 Surgical resilience as a measurable and modifiable construct

Surgical resilience is best conceptualized as a dynamic, multidimensional adaptive process rather than a fixed trait. It reflects the patient's capacity to maintain or regain functional, psychological, and physiological equilibrium in response to surgical stress over time. This process emerges from the interaction between biological reserve, psychological adaptability—including coping strategies, emotional regulation, and self-efficacy—and behavioral engagement, influenced by contextual factors such as social support and access to care (10).

Importantly, resilience does not imply the absence of pain or stress, but rather the ability to sustain meaningful functioning despite these challenges (10). In the surgical context, it can be understood as the capacity to tolerate physiological disruption and return to baseline function following surgical insult (12).

To enhance conceptual clarity, Table 1 summarizes key perspectives on resilience and situates the present framework within existing models.

**Table 1:** Conceptual models of resilience.

Conceptualization	Definition	Key References	Position in Current Model
Trait-based	Stable psychological characteristic	Connor and Davidson (13)	Partial component
Process-based	Dynamic adaptation over time	Amado et al. (10)	Core framework
System-based	Interaction of multiple domains	Graham et al. (12)	Integrated model

**Figure 1:**
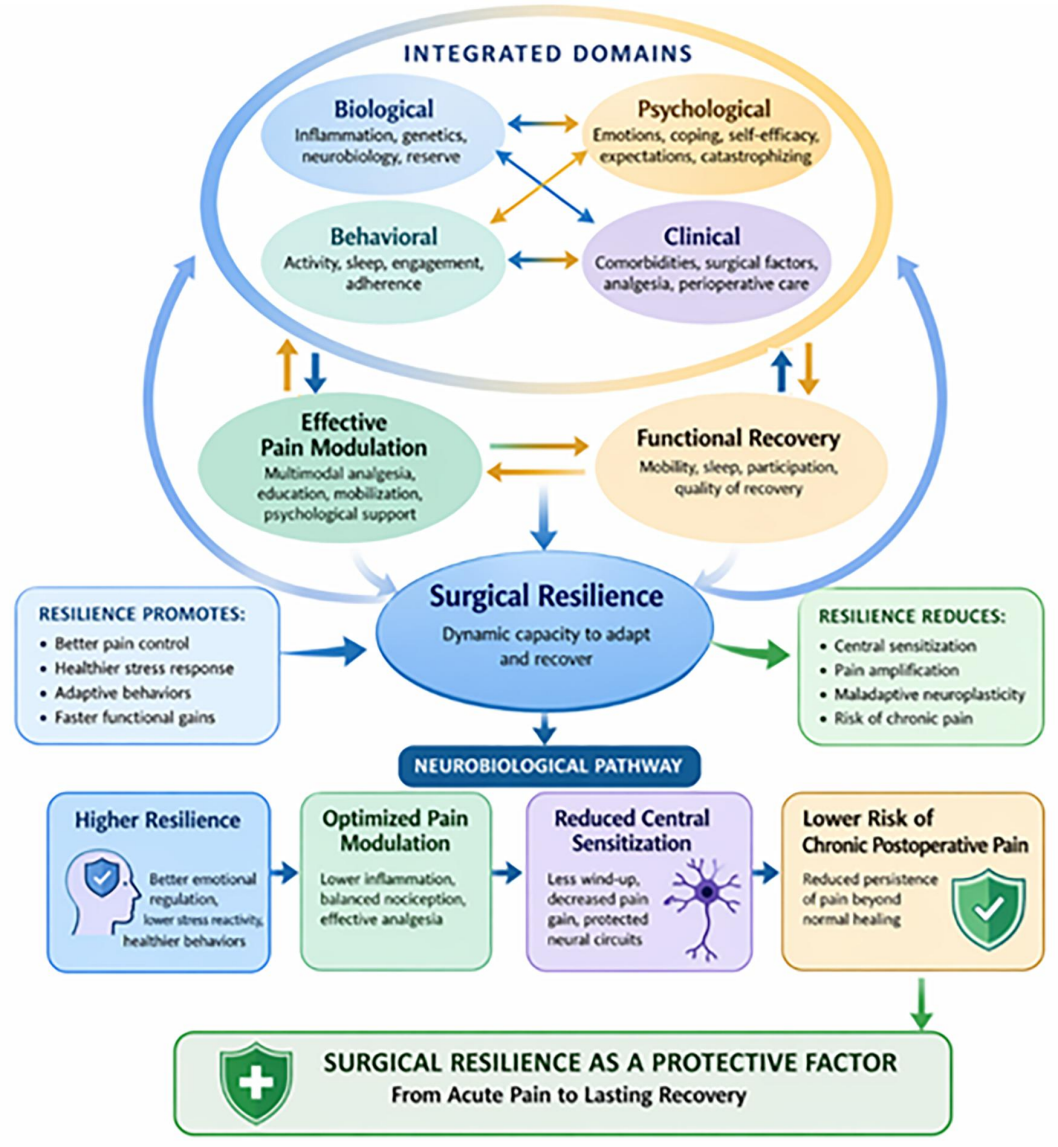
Multidimensional framework of surgical resilience.

This framework provides an integrative model of how perioperative interventions shape recovery trajectories through the modulation of surgical resilience (Figure 1).

Pain modulation acts as a central regulator linking biological, psychological, and behavioral domains. Inadequate modulation may lead to central sensitization, pain catastrophizing, and persistent pain, whereas resilience-enhancing mechanisms promote adaptive recovery, reduce pain interference, and prevent the transition to chronic postsurgical pain.

Note: AI-assisted design tools (Nano Banana and Chat GPT), with full conceptual validation and final approval by the authors.

### 1.2 Psychological and behavioral dimensions of recovery

Social cognitive theory provides a relevant framework for understanding adaptation to surgical stress (14). Self-efficacy—the belief in one's ability to manage challenging situations—directly influences recovery behaviors, including adherence to rehabilitation and engagement in early mobilization (14).

Patients with higher resilience and self-efficacy tend to demonstrate better functional outcomes and lower pain interference (9, 10). Conversely, pain catastrophizing is associated with increased pain perception, reduced engagement, and poorer outcomes (8).

These findings underscore that postoperative recovery is shaped not only by biological processes but also by cognitive and behavioral mechanisms.

### 1.3 Pain as a central modulator of recovery

Within this framework, pain should be understood not merely as a consequence of surgery, but as an active mediator of recovery trajectories.

Inadequate control of acute pain is associated with impaired mobilization, sleep disturbance, respiratory dysfunction, increased risk of complications, and progression to chronic postsurgical pain (15–17). Conversely, effective analgesia attenuates stress responses and facilitates functional recovery (17).

However, the goal of pain management is not the complete elimination of pain at any cost. Excessive reliance on high-dose opioids may impair recovery and increase adverse events, whereas insufficient analgesia limits patient engagement. A resilience-oriented approach therefore seeks to optimize pain control to enable functional recovery, aligning with multimodal and opioid-sparing strategies (17, 18).

### 1.4 Operationalization and measurement

The translation of surgical resilience into clinical practice requires reliable and feasible assessment strategies.

Validated psychometric tools, such as CD-RISC (13) and PROMIS-based instruments, provide structured evaluation of resilience-related domains. These tools are reproducible and sensitive to change; however, important limitations remain, including the lack of perioperative-specific thresholds and variability across populations (10). A summary of available approaches is presented in Table 2.

**Table 2:** Measurement approaches for surgical resilience.

Domain	Tool	Advantages	Limitations	Clinical Use
Psychological	CD-RISC	Validated, widely used	No perioperative cut-offs	Preoperative screening
PROMs	PROMIS, QoR-15	Multidimensional, dynamic	Workflow integration required	Monitoring recovery
Behavioral	Functional engagement	Clinically relevant	Not standardized	Rehabilitation tracking
Biological	NPY, DHEA	Mechanistic insight	Experimental	Research Only
Advanced analytics	ML-based PROMs	Predictive potential	Early-stage	Future integration

Biomarkers such as neuropeptide Y and dehydroepiandrosterone (DHEA) have been proposed as physiological correlates of resilience; however, current evidence remains exploratory and insufficient for routine clinical application (11).

### 1.5 Clinical integration of surgical resilience

The integration of surgical resilience into perioperative care requires structured, clinically actionable strategies across the preoperative, intraoperative, and postoperative phases. Preoperative assessment should include the identification of patients at increased risk for poor recovery and chronic postsurgical pain, using validated instruments such as the Connor–Davidson Resilience Scale (CD-RISC), PROMIS measures, and screening tools for anxiety, depression *(Hospital Anxiety and Depression Scale – HADS)*, and pain catastrophizing *(Catastrophizing Scale – PCS)* (10, 13, 19). In practical terms, resilience screening can be incorporated into the preoperative assessment clinic using brief validated tools (e.g., CD-RISC, BRS), requiring less than 5–10 min to administer. Patients identified as low resilience may be referred to structured perioperative support programs, including brief cognitive-behavioral interventions or resilience training protocols integrated into prehabilitation pathways. Ideally, this evaluation should occur during the preoperative visit, allowing early stratification of vulnerability and timely implementation of targeted interventions.

#### 1.5.1 Preoperative

Interventions aimed at enhancing resilience include cognitive-behavioral strategies, structured patient education, expectation management, psychological prehabilitation, and sleep optimization. These approaches can be incorporated into established perioperative pathways, such as ERAS protocols (6, 19), and should be aligned with individualized care planning.

#### 1.5.2 Intraoperative

Multimodal analgesia remains a central component and should be tailored not only to surgical factors but also to patient-specific risk profiles. The use of regional anesthesia techniques, non-opioid analgesics, and opioid-sparing strategies supports effective pain control, which should be understood as a facilitator of functional recovery rather than an isolated endpoint.

#### 1.5.3 Postoperative

**Figure 2:**
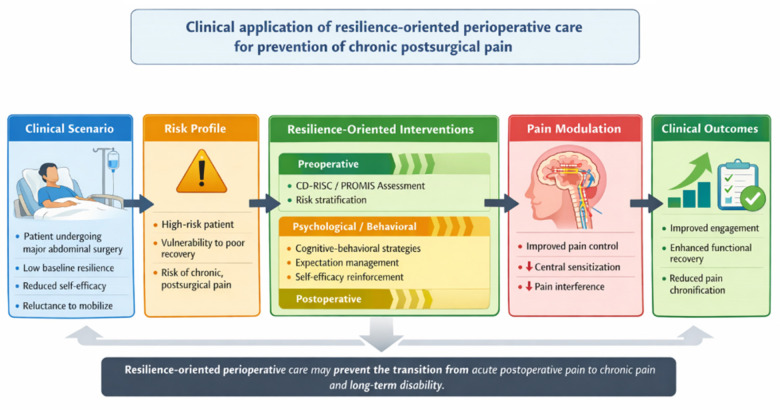
Clinical application of a resilience-oriented perioperative care model for prevention of chronic postsurgical pain.

Postoperative follow-up should incorporate patient-reported outcome measures (PROMs), such as QoR-15 and PROMIS (10), enabling dynamic monitoring of recovery trajectories and supporting individualized, value-based care. Early identification of persistent pain or impaired recovery allows timely intervention, potentially preventing progression to chronic postsurgical pain and long-term disability. This approach reframes perioperative care from a reactive model focused on symptom control to a **proactive strategy aimed at preventing chronic pain and long-term disability through resilience optimization**. The clinical application of this resilience-oriented framework is illustrated in Figure 2.

A high-risk patient undergoing major abdominal surgery is identified through preoperative assessment, including evaluation of resilience and psychosocial factors. Targeted, resilience-enhancing interventions—encompassing cognitive-behavioral strategies, structured patient education, and individualized perioperative planning—are implemented across the perioperative continuum. These interventions optimize pain modulation, reduce central sensitization, and decrease pain interference. As a result, improved patient engagement and functional recovery are achieved, ultimately mitigating the transition from acute postoperative pain to chronic postsurgical pain and long-term disability.

Note: AI-assisted design tools (Nano Banana and Chat GPT), with full conceptual validation and final approval by the authors.

## 2 Discussion

Perioperative medicine is undergoing a clear conceptual shift toward more integrated and patient-centered models of care (20). Despite significant advances in surgical techniques, anesthetic management, and structured pathways such as ERAS, postoperative recovery still varies considerably between patients. Even within optimized protocols, this variability cannot be fully explained by surgical or procedural factors alone (21). This gap highlights the need to better understand the role of individual adaptive capacity in shaping recovery.

In this context, resilience has emerged as an important factor influencing postoperative trajectories. Growing evidence suggests that resilience influences how patients experience pain, engage with recovery, and ultimately regain function and quality of life (9, 10, 22). Recent studies further support this association by demonstrating that higher resilience is linked to reduced pain interference and improved postoperative outcomes, reinforcing its role as a clinically relevant determinant of recovery trajectories (11).

Pain sits at the center of this discussion. When poorly controlled, acute postoperative pain can amplify stress responses, limit mobility, and disrupt recovery, increasing the risk of long-term complications, including chronic postsurgical pain (17, 18, 23). On the other hand, when managed appropriately, pain control supports early mobilization, functional engagement, and a more adaptive recovery process. From this perspective, pain is not just an outcome of surgery, but an active driver of how recovery unfolds.

Much of what we know about resilience in the surgical setting comes from orthopedic populations, where pain and function are key outcomes (9, 10). However, more recent evidence suggests that these findings are not limited to a single specialty. In oncologic and urologic surgery, for example, resilience has been associated with better recovery and improved quality of life, particularly in the weeks and months following surgery, when patients are adapting to ongoing symptoms and stressors (24). Similarly, in major abdominal and high-risk procedures, resilience appears to influence recovery through its effects on stress regulation, coping strategies, and patient engagement, all of which play a role in pain trajectories and functional outcomes (10, 19). Together, these findings suggest that resilience is not a context-specific phenomenon, but a broader, cross-cutting determinant of recovery across surgical disciplines.

From a practical standpoint, resilience can be incorporated into perioperative care in a structured and feasible way. Screening tools such as the CD-RISC or BRS allow clinicians to identify patients who may be at higher risk for poor outcomes, including persistent pain. These patients, in turn, are likely to benefit the most from targeted strategies, such as cognitive-behavioral interventions, resilience training, and enhanced perioperative support (3, 10). An important insight from the literature is that resilience does not need to be maximized to be effective. Instead, the greatest clinical gains appear to come from moving patients out of the lowest resilience range into a more adaptive baseline (8). This has practical implications, suggesting that perioperative interventions can be both focused and efficient.

At the same time, important limitations remain. The lack of standardized definitions continues to make comparisons across studies difficult (10). Measurement tools vary, and clinically meaningful thresholds are not yet well established. In addition, much of the existing evidence comes from single-center observational studies, which limits generalizability. There is still a clear need for larger, multicenter studies and, importantly, for interventional trials that test whether resilience-focused strategies can meaningfully improve outcomes.

Looking ahead, advancing this field will require a more consistent conceptual framework, better tools for clinical assessment, and stronger evidence from longitudinal and pragmatic studies. Integrating psychological, biological, and functional data into predictive models may also help move toward a more personalized approach to perioperative care.

Ultimately, a resilience-oriented perspective offers a practical way to rethink postoperative recovery. Rather than focusing only on controlling pain, it encourages us to support the patient's ability to adapt. In doing so, it opens a path toward reducing the transition from acute postoperative pain to chronic pain and long-term disability.

## 3 Conclusion

Perioperative medicine has traditionally focused on controlling surgical stress and postoperative pain. However, recovery is determined not only by these factors but by the patient's capacity to adapt.

A resilience-oriented framework shifts the focus from passive recovery to active adaptation, integrating biological, psychological, and behavioral domains.

Ultimately, the key question is not whether we control pain, but whether we enable patients to recover—by actively supporting their capacity to adapt, engage, and restore function in the face of surgical stress.

### Take-home messages

- Resilience-oriented perioperative care enhances patient engagement, reduces pain interference, and improves functional recovery.
- Early identification of high-risk patients enables targeted, multimodal interventions across the perioperative continuum.
- Optimizing resilience may prevent the transition from acute postoperative pain to chronic pain and long-term disability.

## Data Availability

The original contributions presented in the study are included in the article/Supplementary Material, further inquiries can be directed to the corresponding author.
